# Effect of surface ligands on the optical properties of aqueous soluble CdTe quantum dots

**DOI:** 10.1186/1556-276X-7-536

**Published:** 2012-09-27

**Authors:** Fernanda O Silva, Melissa S Carvalho, Renato Mendonça, Waldemar AA Macedo, Karla Balzuweit, Peter Reiss, Marco A Schiavon

**Affiliations:** 1 Grupo de Pesquisa em Química de Materiais - (GPQM), Departamento de Ciências Naturais, Universidade Federal de São João Del Rei, Campus Dom Bosco, Praça Dom Helvécio, 74, CEP, São João Del Rei, Minas Gerais, 36301-160, Brazil; 2 Laboratório de Física Aplicada, Centro de Desenvolvimento da Tecnologia Nuclear, Belo Horizonte, Minas Gerais, 31270-901, Brazil; 3 Departamento de Física, ICEx, Universidade Federal de Minas Gerais, Belo Horizonte, Minas Gerais, 30123-970, Brazil; 4 CEA-Grenoble, INAC-SPrAM (UMR 5819 CEA-CNRS-UJF)-LEMOH, 17 Rue des Martyrs, 38054, Grenoble, Cedex 9, France

**Keywords:** CdTe, Surface ligands, Optical properties, Semiconductor nanocrystals

## Abstract

We investigate systematically the influence of the nature of thiol-type capping ligands on the optical and structural properties of highly luminescent CdTe quantum dots synthesized in aqueous media, comparing mercaptopropionic acid (MPA), thioglycolic acid (TGA), 1-thioglycerol (TGH), and glutathione (GSH). The growth rate, size distribution, and quantum yield strongly depend on the type of surface ligand used. While TGH binds too strongly to the nanocrystal surface inhibiting growth, the use of GSH results in the fastest growth kinetics. TGA and MPA show intermediate growth kinetics, but MPA yields a much lower initial size distribution than TGA. The obtained fluorescence quantum yields range from 38% to 73%. XPS studies unambiguously put into evidence the formation of a CdS shell on the CdTe core due to the thermal decomposition of the capping ligands. This shell is thicker when GSH is used as ligand, as compared with TGA ligands.

## Background

Over the past two decades, semiconductor nanocrystals have attracted great attention of researchers due to their unique optical properties. In particular, luminescent quantum dots (QDs) are defined as semiconductor structures with physical dimensions that are smaller than the exciton Bohr radius [1,2]. These materials exhibit a strong quantum confinement effect, and this effect causes the appearance of size-dependent optical properties, which has attracted great attention for application of QDs in different technological areas, including biological labeling, light-emitting diodes, and photovoltaic devices [3].

QDs can be produced via a number of synthetic methods. These techniques have great advances in recent years, which have enabled the synthesis of monodisperse nanocrystals with good optical properties as well as different compositions and morphologies [4]. Up to now, the most successful method to prepare highly luminescent II-VI colloidal semiconductors is the organometallic synthetic route, which uses trioctylphosphine oxide and trioctylphosphine as surface ligands, in order to avoid nanocrystal growth and aggregation. Alternatively, organic metal salts such as Cd carboxylates or phosphonates can be reacted with the chalcogenide source in 1-octadecene. However, these methods require high temperatures, and the resulting nanoparticles are insoluble in water, which makes the final product incompatible with the biological systems [5-7]. More recently, QDs have been prepared in aqueous medium because this synthetic approach is simpler, less toxic, and generates water-soluble nanocrystals that are directly biologically compatible [8]. Nevertheless, this method generally produces nanoparticles with lower fluorescence quantum yields, when compared to the synthesis in organic media. The lower florescence quantum yield is attributed to the defects and traps on the surface of the nanocrystals. Therefore, researchers have investigated the influence of the surface ligands so as to remove these defects and improve the optical properties [4,9,10].

Surface ligands consist of a polar anchoring group and either an apolar hydrocarbon chain (synthesis in organics) or a charged group (synthesis in water). These ligands must dynamically adsorb on/desorb from the surface of the nanocrystals at the synthesis temperature in order to allow for growth while the nanoparticles are stabilized against aggregation. Peng et al*.* reported on the effect of amine ligands on the growth of CdSe QDs and proved that the ligand dynamics on the nanocrystal surface depends on the reaction temperature and on the concentration and chain length of the stabilizers [11]. Earlier studies showed that ligands play an important role during the formation of nanocrystals, exerting a strong effect on both the nucleation and growth stages. Hence, surface ligands can control the size, shape, growth kinetics, and optical properties of the QDs [4,10-14].

Also, in the case of water-soluble CdTe nanocrystals, the influence of some types of surface ligands on the structural and optical properties has been studied [15,16]. The most often used surface ligands are thioglycolic acid (TGA) or mercaptopropionic acid (MPA). The growth kinetics of TGA-coated CdTe has been analyzed quantitatively by means of dynamic light scattering (DLS) measurements, and the growth rates, size distributions, critical radii, and diffusion constants have been calculated in the framework of the theoretical Sugimoto model [17]. There are two distinct regimes of kinetics: (1) slow increase in the hydrodynamic radius and (2) faster growth of nanoparticles compared with the previous regime. These two kinetic regimes allow for a certain control of nanocrystal size and size distribution, with no need for post-preparative fractionation techniques.

In 2010, Lesnyak et al. [18] described a novel ligand, 5-mercaptomethyltetrazole, for the aqueous synthesis of CdTe nanocrystals. Tetrazoles are five-membered cyclic compounds containing four nitrogen atoms of different types (pyrrole and pyridine type). CdTe nanocrystals obtained via the ‘standard method’ but using mercaptomethyltetrazole instead of TGA as the stabilizer exhibited fluorescence in the 510- to 610-nm range, depending on the reflux time, and fluorescence quantum yields reaching up to 60%. Upon addition of a solution of Cd^2+^ ions, the CdTe nanocrystals irreversibly formed hydrogels, i.e., highly porous 3D networks [18]. The interaction of MPA-capped CdTe nanocrystals, synthesized in aqueous media with cysteine and homocysteine, has already been described [19]. Glutathione (GSH), a thiol-containing tripeptide, has been shown to be able to provide improved biocompatible capping for semiconductor nanocrystals as compared with many other water-soluble ligands [20]. Moreover, GSH appears to work best with CdTe in terms of promoting high photoluminescence [10,21].

In the present work, we conducted a comparative study of the synthesis of CdTe QDs prepared in aqueous media using four different surface ligands: MPA, TGA, 1-thioglycerol (TGH), and GSH. The influence of these ligands on the surface of the nanocrystals was evaluated on the basis of the changes in their optical properties and their sizes.

## Methods

### Chemicals

Tellurium powder (200 mesh, 99.8%), TGH (98%), reduced l-glutathione (GSH, 99%), 3-MPA (99%), and rhodamine 101 (100%) were obtained from Sigma-Aldrich (St. Louis, MO, USA). TGA (99%) and thiourea (99%) were purchased from Synth (Diadema, São Paulo, Brazil). Sodium borohydride (NaBH_4_, 97%) was acquired from Nuclear (São Paulo, Brazil), and cadmium chloride monohydrate (CdCl_2_·H_2_O, 99%) was provided by Vetec (Rio de Janeiro, Brazil). All the chemicals were used without additional purification. Milli-Q ultrapure water (Millipore Co., Billerica, MA, USA) was employed in the synthesis of nanoparticles.

### Syntheses of MPA-, TGA-, TGH- and GSH-coated CdTe QDs

The experimental procedure was performed according to [22], but different stabilizing ligands were utilized. Briefly, 0.8 mmol of tellurium powder and 1.6 mmol of sodium borohydride were diluted in 20 mL of Milli-Q water in a 25-mL three-neck flask. The reaction mixture was heated to 80°C under argon flow in order to obtain a clear deep red solution. The resulting NaHTe was used as tellurium source. Next, 0.4 mmol of Cd solution and 1.4 mmol of MPA were mixed in 80 mL of Milli-Q water, and the pH value was adjusted to 10.0 by the addition of NaOH (1.0 molL^−1^). This solution was heated at 100°C under argon bubbling and then 4.0 mL of freshly prepared NaHTe was added with the aid of a syringe. The resulting solution was refluxed at 100°C for different times in order to obtain CdTe QDs of different sizes. Aliquots were taken at defined time intervals, and their UV-vis absorption and photoluminescence (PL) spectra were recorded. Samples were precipitated by addition of acetone and dried in vacuum prior to characterization by X-ray diffration (XRD), Fourier-transform infrared spectroscopy (FTIR), X-ray photoelectron spectroscopy (XPS), and transmission electron microscopy (TEM). The same procedure was followed for the synthesis involving the other surface ligands investigated in this work. For acquisition of the TEM images, the nanocrystals were submitted to a phase-transfer procedure based on the partial exchange of the MPA stabilized by dodecanethiol (DDT) [23]. To this end, 1 mL of an aqueous solution of CdTe was placed in a test tube, and 1 mL of 1-dodecanethiol and 2 to 3 mL of acetone were added to this solution. The test tube was vigorously shaken and heated to the boiling point of acetone. The transfer of the nanocrystals to the organic phase was detected from the change in the color of the latter phase.

### Characterization

UV-vis absorption and PL spectra were acquired on a Varian Cary 50 spectrophotometer (Varian Inc., Palo Alto, CA, USA) and Shimadzu RF-5301 PC spectrofluorophotometer (Shimadzu Corporation, Nakagyo-ku, Kyoto, Japan), respectively. The spectrofluorophotometer is equipped with a xenon lamp of 150 W. The absorption and fluorescence measurements were typically performed with 10-mm quartz cuvettes (Shimadzu) using air-saturated solutions at room temperature. The fluorescence quantum yield (*ϕ*_f_) of the nanocrystals was estimated by comparing the integrated emission of the QD samples, obtained at one excitation wavelength, with that of a standard fluorescent dye, rhodamine 101 [24,25]. We have used the wavelength excitation of 355 nm. Essentially, stock solutions of the standard and QD samples with similar absorbance (no higher than 0.02) at the same excitation wavelength can be assumed to be absorbing the same number of photons. Hence, a simple ratio of the integrated fluorescence intensities of the two solutions (recorded under identical conditions) yielded the ratio of the quantum yield values. Since the quantum yield for the standard sample rhodamine 101 is known (*ϕ*_f_ = 1.0 in water [24,25]), it is trivial to calculate the quantum yield for the QDs. Identical instrument settings for the sample and standard solutions were carefully checked, and the solvent adsorption and emission spectra were subtracted from the absorption and emission spectra of the sample and standard solutions. This was done directly in the software of the equipment used. In addition, the measurements were repeated for at least three different concentrations of the sample and the reference dye. Powder X-ray diffraction (XRD) patterns were recorded on a Shimadzu XRD-6000 using CuK*α* radiation. FTIR spectra of the materials were obtained by the conventional KBr pellet technique, in a GXI spectrum Perkin Elmer spectrometer (PerkinElmer, Waltham, MA, USA), operating between 4,000 and 400 cm^−1^. A minimum of 32 scans were recorded with a resolution of 2 cm^−1^. The KBr salt and the nanoparticles were dried for 2 h under 110°C and 40°C, respectively. Both samples were kept under vacuum until the moment of the analyses. The sizes of the nanocrystals were determined by DLS measurements using a HeNe source (*λ* = 632.8 nm) and a photomultiplier as detector. The correlation functions were calculated by the BI9000-AT correlator board Brookhaven Inst. Co. (Holtsville, NY, USA). A scattering angle of 30.0° was used during these measurements, and the samples were maintained in a thermal bath at 25.0°C with a precision of 0.1°C. TEM was performed on a TEM-FEG JEM 2100F microscope (JEOL Ltd., Akishima, Tokyo, Japan) operating at 200 kV. XPS was conducted on an ultrahigh vacuum system (base pressure of 3.0 × 10^−10^ mbar) equipped with a standard non-monochromatic Mg Kα X-ray source (hν = 1,253.6 eV) and a concentric hemispherical electron-energy analyzer (CLAM2, VG Microtech, East Sussex, UK). The binding energy (BE) scale was calibrated using the carbon peak from the surface contamination as reference (C 1*s* at 284.6 eV).

## Results and discussion

**Figure 1:**
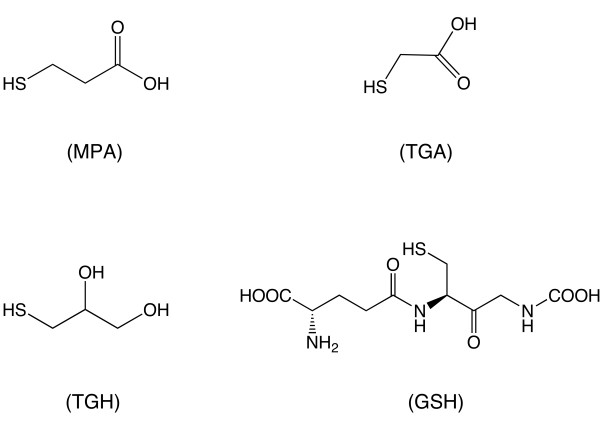
Schematic representation of the molecular structures of the surface ligands investigated in this work.

There are many studies reporting on the synthesis of CdTe QDs, but only few of them have described the influence of surface ligands on the growth and optical properties of these nanocrystals. In this work, we investigated the effect of for different thiol ligands on the growth and optical properties of CdTe nanocrystals prepared under identical synthesis conditions. The molecular structures of these ligands are schematically represented in Figure 1.

**Figure 2:**
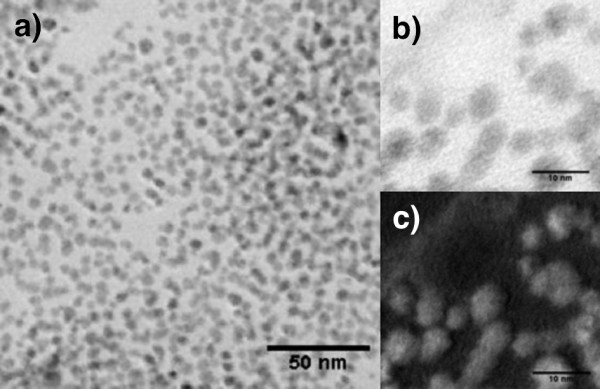
**TEM images. **(**a**) TEM image of CdTe NCs transferred into toluene after exchange of the stabilizing MPA ligands with DDT; (**b**) TEM bright field image; and (**c**) TEM HAADF image.

Representative STEM images of the obtained CdTe QDs are displayed in Figure 2. These images show that the nanocrystals have approximately spherical shape and are well dispersed without aggregation.

**Figure 3:**
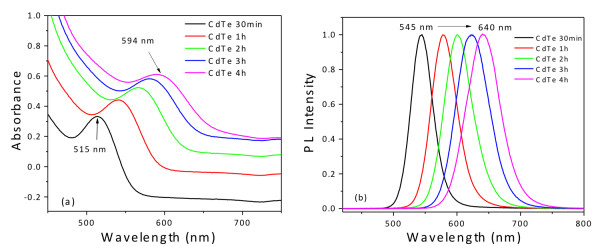
**Temporal evolution. **(**a**) The UV-vis absorption and (**b**) the PL emission spectra of GSH-capped CdTe QDs (excitation wavelength 355 nm).

**Figure 4:**
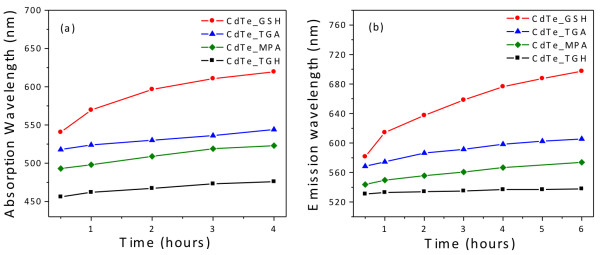
**Temporal evolution. **(**a**) The absorption and (**b**) the PL peak position of CdTe QDs capped with different surface ligands.

Figure 3 illustrates the temporal evolution of the UV-vis absorption and PL spectra of GSH-coated CdTe QDs. A longer refluxing time systematically shifts the excitonic absorption and PL emission peaks to longer wavelengths, which is a clear indication of nanocrystal growth. This behavior is different from that verified for the other investigated ligands. Figure 4 presents the temporal evolution of the absorption and emission peaks recorded for the four surface ligands studied here. The growth rate registered for GSH-coated CdTe QDs is the highest under the same refluxing conditions. We attribute the special behavior of GSH to its higher steric hindrance and its stronger tendency to thermal decomposition as compared to the other ligands. Under reflux, the partial hydrolysis of GSH causes the incorporation of sulfur in the interior and surface of the CdTe nanocrystals, thereby forming a CdTe/CdS core/shell or a graded structure [26-29]. The latter induces spectral shifts but also provides better surface passivation and control over defects, improving the fluorescence quantum yield. As for the other three ligands, the growth rates of CdTe QDs capped with are significantly lower, suggesting that they bind more strongly to the surface of the nanocrystal. While the temporal evolution of the peak positions is similar for all the three ligands, the size of the initially formed crystallites is smallest for TGH, followed by MPA and TGA.

**Figure 5:**
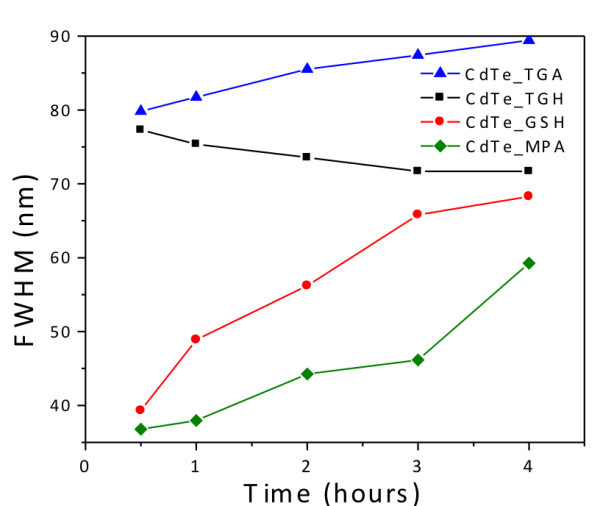
PL FWHM of CdTe QDs capped with different surface ligands.

In Figure 5, the PL linewidth of the QDs (full width at half-maximum (FWHM)) is plotted as a function of the reaction time. As a rule of thumb, the lower the linewidth, the narrower is the size dispersion. Contrary to the synthesis in organic solvents, no size focusing is observed [30]. For all reaction times, MPA-coated CdTe QDs have the narrowest size distribution followed by GSH-coated ones. In both cases, a broadening of the linewidth is detected with longer reflux time. Both TGA- and TGH-coated CdTe yield a much broader initial linewidth. This behavior indicates that these ligands bind too strongly to the surface of the nanocrystal, avoiding the sharp separation of nucleation and growth under the reaction conditions employed here. As for TGH, the linewidth becomes slightly narrower with reaction time, while a further broadening is verified in the case of TGA. Broad size distributions can be reduced using the post-preparative size-selective precipitation technique [22].

**Figure 6:**
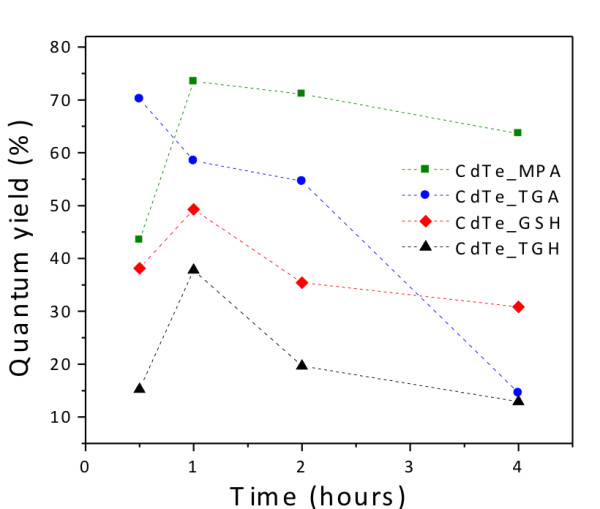
**Quantum yield ( *ϕ ***_**f**_**) of CdTe QDs capped with different surface ligands. ** Dotted lines are added to guide the eye.

Figure 6 depicts the *ϕ*_f_ of the as-synthesized CdTe QDs at different reaction times. The maximal quantum yields are achieved after 1 h of synthesis (MPA 73%, GSH 49%, and TGH 38%), except for TGA, which reached the maximum after half an hour (70%). This demonstrates that short-chain ligands, such as MPA and TGA, generally give rise to high *ϕ*_f_, which is likely due to a better surface passivation induced by a higher ligand density [9]. Under prolonged reflux, there is a decrease in *ϕ*_f_ values for all ligands. The fluorescence quantum yield is strongly dependent on the surface quality. At longer reflux times, unfavorable adsorption-desorption equilibria associated with the Ostwald ripening phenomenon can lead to incomplete passivation of surface trap states of the QDs, resulting in lower fluorescence quantum yield [31]. Rogach et al. [8] have proposed a correlation between the values of *ϕ*_f_ and Stokes shift as a rapid technique to evaluate the quality of the samples, without involving the comparison with luminescence standards. In brief, high *ϕ*_f_ samples generally exhibit a lower Stokes shift than low *ϕ*_f_ samples. In the former case, detrapping of carriers from shallow trap levels occurs, while in the latter case, a broad distribution of trap states favors non-radiative de-excitation. A comparison of the *ϕ*_f_ values and Stokes shift of CdTe-capped with GSH, TGA, MPA, and TGH at different synthesis times is summarized in Table 1. For the smaller molecules (TGA, MPA, and TGH), we observe indeed the general trend that a decrease in the *ϕ*_f_ value is accompanied by an increase of Stokes shift. In particular, CdTe-TGH show the lowest *ϕ*_f_ values combined with the highest Stokes shifts. On the other side, CdTe QDs capped with the larger molecule GSH display a different behavior; although the lowest Stokes shift is observed, only intermediate *ϕ*_f_ values are measured. The lower Stokes shift indicates that deep traps are efficiently passivated by a thin, *in situ*-generated ZnS shell on the CdTe core (vide infra). At the same time, *ϕ*_f_ is probably reduced by a variety of non-radiative de-excitation pathways resulting from the insufficient passivation of (outer) surface states in case of the sterically demanding GSH ligands.

**Table 1:** **Comparison of*ϕ***_**f **_**and Stokes shift of CdTe QDs**

**Synthesis time**	**GSH**		**TGA**		**MPA**		**TGH**	
	***ϕ***_**f**_**(%)**	**Stokes shift (meV)**	***ϕ***_**f**_**(%)**	**Stokes shift (meV)**	***ϕ***_**f**_**(%)**	**Stokes shift (meV)**	***ϕ***_**f**_**(%)**	**Stokes shift (meV)**
30 min	38	162	70	289	44	222	15	380
1 h	49	160	58	211	73	231	38	353
2 h	35.5	134	54.5	224	71	207	20	329
4 h	31	169	14.5	206	63.5	180	13	297

CdTe QDs capped with GSH, TGA, MPA, and TGH at different synthesis times.

**Figure 7:**
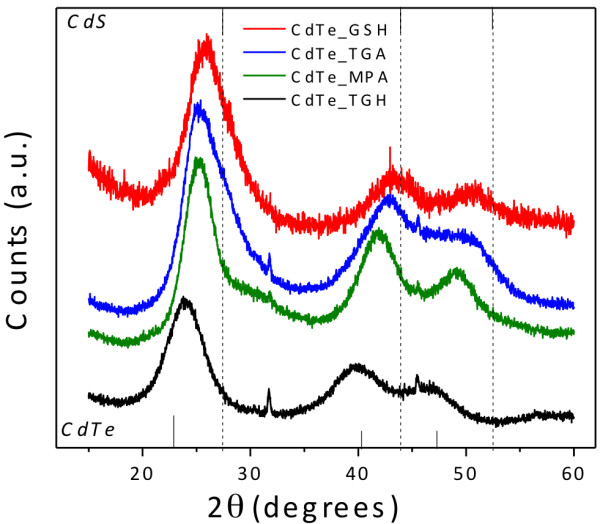
XRD patterns of CdTe QDs capped with different surface ligands.

The XRD patterns of CdTe powders precipitated from aqueous sols of QDs with an excess of acetone are given in Figure 7. The nanocrystals belong to the cubic zinc-blende structure with diffraction peaks at 24°, 40°, and 46.7°, which is consistent with the dominant crystal phase of bulk CdTe [32]. However, when GSH is used as surface ligand, the diffraction peaks shift to larger angles toward the peaks of the CdS. As mentioned before, the thermal decomposition of GSH favors the formation of CdTe/CdS core/shell or graded structures. TGH-coated CdTe nanocrystals, on the other hand, undergo a smaller shift toward the peak positions of CdS, which proves that there is smaller tendency for the formation of CdTe/CdS structures. Finally, TGA and MPA exhibit intermediate peak positions between those of cubic CdTe and CdS.

**Figure 8:**
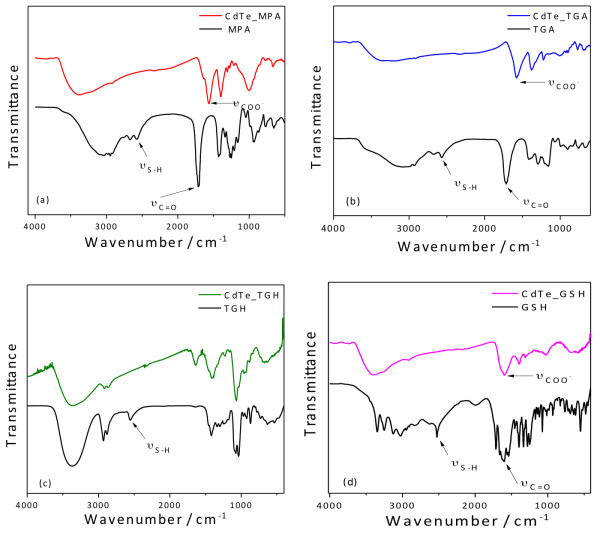
Infrared transmission spectra of (a) MPA-, (b) TGA-, (c) TGH-, and (d) GSH- capped CdTe QDs.

Figure 8 presents the FTIR spectra of the nanocrystals stabilized with different ligands. The main differences of the spectra between the free and bound ligands are marked with arrows. In the case of free TGA and MPA, the most pronounced IR absorption bands occur at 3,500 to 3,000 cm^−1^ ( $\upsilon_{\text{OH}}$), 2,950 cm^−1^ ( $\upsilon_{{\text{CH}}_{2}}$), 2,574 cm^−1^ ( $\upsilon_{\text{SH}}$), 1,707 cm^−1^ ( $\upsilon_{\text{C}=\text{O}}$), 1,222 cm^−1^ ( $\upsilon_{\text{C}-\text{O}}$), and 680 cm^−1^ ( $\delta_{\text{C}-\text{S}}$). For the bound ligands, the COO^−^ vibrations at 1,562 cm^−1^ and 1,397 cm^−1^ are consistent with the fact that at pH 10, the carboxylic acid group is deprotonated given its pK_COOH_ value of 3.67. The S-H vibrations (2,574 cm^−1^) are not detectable in the IR spectra of any of the bound ligands, which is expected for thiols covalently bound to the surface of nanocrystals.

The hydrodynamic size of the nanocrystals has been measured by dynamic light scattering [33-35]. This technique allows for the statistical analysis of the fluctuations in the intensity of the light scattered by particles in solutions. The fluctuations in the intensity of the scattered light are expected to undergo exponential decay along time, at a rate *Γ* given by the product of the diffusion coefficient *D* and the squared scattering wave vector *q*, which is defined in terms of the wavelength *λ* of the source, the refraction index *n,* and the scattering angle *θ:*

(1) $$\Gamma =Dq^{2}=D{\left[\left(4\pi n/\lambda \right)\text{sin}\left(\theta /2\right)\right]}^{2}\text{.}$$

The Einstein-Stokes relation is then used for calculation of the hydrodynamic radius of the dispersed particles from the diffusion coefficient *D*, the solvent viscosity *η*, and the thermal energy *k*_*B*_*T*:

(2) $$R_{\text{h}}=k_{B}T/6\pi \eta D\text{.}$$

The intensity correlation function *C*(*q*,*t*) is proportional to the squared dynamic structure factor *S*(*q*,*t*) ( $C\left(q,t\right)≅S{\left(q,t\right)}^{2}≅\text{exp}\left(-2\Gamma t\right)$). In the case of bidispersions, *S*(*q*,*t*) becomes the sum of two exponentials corresponding to each species. The measurements can then be well fitted by an expression given by the sum of a fast decaying exponential and a slower one. The decay rate *Γ* is computed from this fitting of the experimental results, and the diffusion coefficient is calculated through Equation 1. This coefficient can be replaced directly in Equation 2 yielding the hydrodynamic radius *R*_h_.

**Figure 9:**
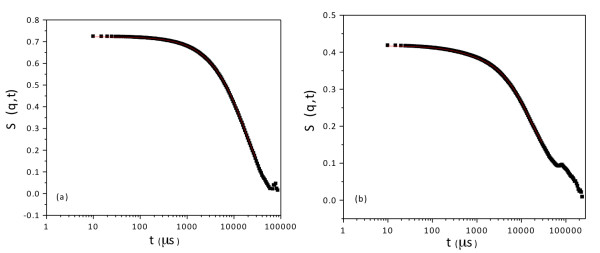
Lin-log correlograms of (a) MPA- and (b) GSH-capped CdTe QDs after 1 h of synthesis.

**Figure 10:**
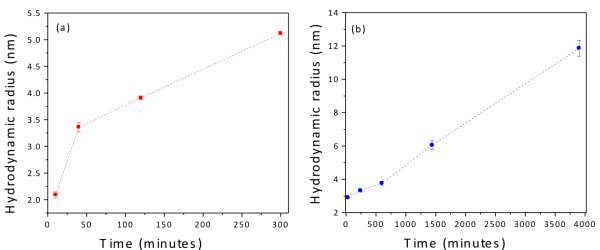
**Hydrodynamic radii (nm) of (a) GSH- and (b) MPA-capped CdTe QDs. ** Dotted lines are added to guide the eye.

Using this technique describe above, we obtained the dynamic structure factor, *S*(*q*,*t*), for the MPA- and GSH-coated CdTe QDs (Figure 9). Following the aforementioned steps, the data shown in Figure 9a,b allow for the determination of the hydrodynamic radii of the nanocrystals. We calculate the hydrodynamic radii of the nanocrystals at different reaction times (Figure 10). The GSH-coated CdTe QDs (Figure 10a) and the MPA-coated CdTe QDs (Figure 10b) have sizes ranging from 2.1 to 5.1 nm and from 2.1 to 11.9 nm, respectively. The large sizes determined for MPA ligands can be ascribed to aggregation occurring at extended reflux times. Indeed, here, the reaction with MPA was conducted for more than 60 h vs. 5 h in the case of GSH. The DLS results also confirm that the growth rate of GSH-capped CdTe QDs is higher than that of MPA-capped QDs under the same refluxing conditions. Nanocrystals with sizes of 6 nm were obtained after approximately 5 and 24 h of synthesis for the GSH and MPA ligands, respectively.

**Figure 11:**
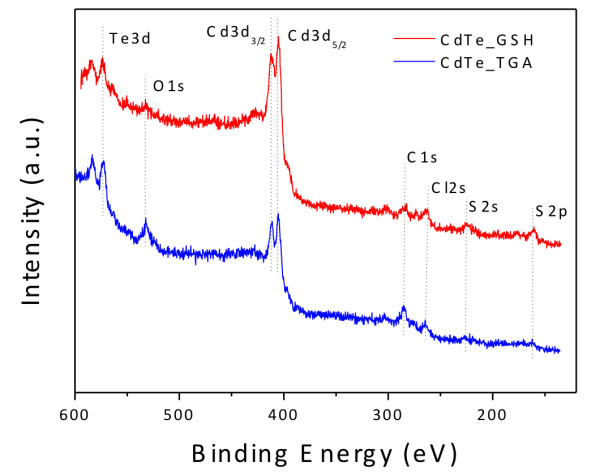
XPS spectra of the GSH- and TGA-capped CdTe QDs.

Finally, we examined the surface of the TGA- and GSH-coated CdTe QDs using XPS. The survey XPS spectra of TGA- and GSH-coated CdTe QDs are displayed in Figure 11. The spectra are characterized by the Cd 3d_5/2_ peaks at 405.0 and 404.8 eV as well as by the Te 3d_5/2_ peaks at 572.9 and 572.6 eV for TGA- and GSH-coated CdTe QDs, respectively. The BE values observed for Te 3d_5/2_ are characteristic of CdTe [36], but the values obtained for Cd 3d_5/2_ are closer to the ones typical of CdS (405.3 eV [36]). The photoemission spectra also reveal the C 1*s* and O 1*s* peaks for the TGA- and GSH-coated CdTe QDs. Comparison between the spectra of both TGA- and GSH-coated CdTe QDs shows that there are higher carbon and oxygen contents in the spectrum of the sample using TGA. Higher sulfur contents (S 2*s* and S 2*p*) can be verified in the spectrum of GSH-coated CdTe QDs, corroborating the hypothesis that the decomposition of this ligand increases the amount of sulfur in the surface layers of the QDs. The S 2*p* peaks are centered at 162.5 and 162.0 eV in the spectra of TGA- and GSH-coated CdTe, respectively, consistent with the formation of CdS [4,37]. Thus, on the basis of the data presented above, we can infer that the decomposition of GSH leads to the formation of a CdS shell on the CdTe core. The higher amounts of cadmium and sulfur in the spectrum of GSH-coated CdTe indicate the formation of a thicker CdS shell than in the case of TGA-coated CdTe. Similar results have been reported for CdTe/CdS core/shell QDs synthesized using tiopronin and thioacetamide, where evidence for the formation of the core/shell structure was obtained from the blueshift of the S 2*p* peak by 0.4 eV from CdTe to CdTe/CdS [4].

## Conclusions

In this work, we carried out a systematic investigation of different thiol-stabilizing ligands on the properties of CdTe QDs synthesized in aqueous solution. The growth rate, size distribution, and quantum yield strongly depend on the type of surface ligand. Under the same refluxing conditions, the highest growth rate is obtained in the presence of the GSH ligand. However, TGH ligands bind to the QD surface too strongly, hindering particle growth and yielding broad size distribution. TGA and MPA ligands furnish comparable results due to their similar molecular structures. The exception is the size dispersion at short reaction times, which is much broader for TGA. No size focusing is observed in any case. With the exception of TGH-coated CdTe QDs, for which a slight decrease in the PL linewidth is observed with reflux time, the QDs exhibit broader size distributions and lower quantum yields for prolonged reaction times. The infrared spectra indicate that the ligands are connected to the nanocrystal surface via the SH group. XPS results clearly evidence the formation of a CdS shell on the CdTe core due to the thermal decomposition of the surface ligands. This CdS shell is thicker when GSH is used as the capping ligand as compared with TGA. In most cases, the shell thickness is directly correlated to the photostability of the QDs, and therefore, GSH-coated CdTe QDs are the most promising candidates for applications relying on good photoluminescence properties such as biological labeling or displays and lighting.

## Competing interests

The authors declare that they have no competing interests.

## Authors' contributions

FOS and MSC carried out the preparation of CdTe samples and drafted the manuscript. RM and WAAM worked on XPS measurements and preparation of the manuscript. KB helped with TEM and the characterization of the samples. PR and MAS participated in the preparation, revision, and finalization of the manuscript. All authors read and approved the final manuscript.
